# Illuminating links between cis-regulators and trans-acting variants in the human prefrontal cortex

**DOI:** 10.1186/s13073-022-01133-8

**Published:** 2022-11-24

**Authors:** Shuang Liu, Hyejung Won, Declan Clarke, Nana Matoba, Saniya Khullar, Yudi Mu, Daifeng Wang, Mark Gerstein

**Affiliations:** 1 Waisman Center, University of Wisconsin – Madison, Madison, WI 53705 USA; 2 Department of Genetics, University of North Carolina at Chapel Hill, Chapel Hill, NC 27599 USA; 3 Neuroscience Center, University of North Carolina at Chapel Hill, Chapel Hill, NC 27599 USA; 4 Department of Molecular Biophysics and Biochemistry, Yale University, New Haven, CT 06520 USA; 5 Department of Biostatistics and Medical Informatics, University of Wisconsin – Madison, Madison, WI 53706 USA; 6 Department of Statistics, University of Wisconsin – Madison, Madison, WI 53706 USA; 7 Department of Computer Sciences, University of Wisconsin – Madison, Madison, WI 53706 USA; 8 Program in Computational Biology and Bioinformatics, Yale University, New Haven, CT 06520 USA; 9 Department of Computer Science, Yale University, New Haven, CT 06520 USA; 10 Department of Statistics and Data Science, Yale University, New Haven, CT 06520 USA

## Abstract

**Background:**

Neuropsychiatric disorders afflict a large portion of the global population and constitute a significant source of disability worldwide. Although Genome-wide Association Studies (GWAS) have identified many disorder-associated variants, the underlying regulatory mechanisms linking them to disorders remain elusive, especially those involving distant genomic elements. Expression quantitative trait loci (eQTLs) constitute a powerful means of providing this missing link. However, most eQTL studies in human brains have focused exclusively on cis-eQTLs, which link variants to nearby genes (i.e., those within 1 Mb of a variant). A complete understanding of disease etiology requires a clearer understanding of trans-regulatory mechanisms, which, in turn, entails a detailed analysis of the relationships between variants and expression changes in distant genes.

**Methods:**

By leveraging large datasets from the PsychENCODE consortium, we conducted a genome-wide survey of trans-eQTLs in the human dorsolateral prefrontal cortex. We also performed colocalization and mediation analyses to identify mediators in trans-regulation and use trans-eQTLs to link GWAS loci to schizophrenia risk genes.

**Results:**

We identified ~80,000 candidate trans-eQTLs (at FDR<0.25) that influence the expression of ~10K target genes (i.e., “trans-eGenes”). We found that many variants associated with these candidate trans-eQTLs overlap with known cis-eQTLs. Moreover, for >60% of these variants (by colocalization), the cis-eQTL’s target gene acts as a mediator for the trans-eQTL SNP's effect on the trans-eGene, highlighting examples of cis-mediation as essential for trans-regulation. Furthermore, many of these colocalized variants fall into a discernable pattern wherein cis-eQTL’s target is a transcription factor or RNA-binding protein, which, in turn, targets the gene associated with the candidate trans-eQTL. Finally, we show that trans-regulatory mechanisms provide valuable insights into psychiatric disorders: beyond what had been possible using only cis-eQTLs, we link an additional 23 GWAS loci and 90 risk genes (using colocalization between candidate trans-eQTLs and schizophrenia GWAS loci).

**Conclusions:**

We demonstrate that the transcriptional architecture of the human brain is orchestrated by both cis- and trans-regulatory variants and found that trans-eQTLs provide insights into brain-disease biology.

**Supplementary Information:**

The online version contains supplementary material available at 10.1186/s13073-022-01133-8.

## Background

Psychiatric disorders exact an immense public health toll on a global scale. Despite the identification of many variants associated with these disorders through genome-wide association studies (GWAS), the precise mechanisms by which they influence human health remain poorly characterized. Expression quantitative trait loci (eQTLs) are often used to analyze the effects of variants on gene expression. An eQTL consists of a variant (termed an “eSNP”) and a gene (termed an “eGene”) wherein the genotype at the eSNP locus is significantly associated with changes in the expression of the eGene. Previous studies have demonstrated that variants associated with a range of human phenotypes frequently function as eQTLs [1], suggesting that eGene expression may play the role of a so-called intermediate phenotype. By definition, however, eQTLs are identified by statistical associations (i.e., correlations), whereas a more complete understanding of disease susceptibility and etiology entails better characterization of causal relationships. By investigating mediation effects in the context of eQTLs, we can more confidently establish such causal relationships.

Although cis-eQTLs are identified for eSNP-eGene pairs that lie within one chromosomal Mb of one another, it is now well established that cis-eSNPs may simultaneously be associated with the expression of distant genes [2, 3]. A natural model for such phenomena may be one in which the expression of a trans-eGene (i.e., the eGene associated with a *distant* eSNP) is mediated by a cis-eGene [3, 4]. Trans-eQTLs that are mediated by cis-eGene expression provide more direct causal relationships between variants and trans-eGene expression. A simple but illustrative example of this phenomenon may involve an eSNP that lies within the promoter of a cis-eGene, wherein the cis-eGene is a transcription factor (TF). The distal target of this TF may then appear as a trans-eGene, the expression of which is strongly influenced by the variant affecting the expression of the regulator TF. Thus, the regulatory linkage in this example comprises an eSNP that influences a nearby TF as part of a cis-eQTL, the cis-eGene of which (the TF) then functions as a mediator that influences the expression of the regulated trans-eGene. Elucidating the roles of mediators such as TFs, RNA-binding proteins, microRNAs, chromosomal remodeling proteins, and other regulatory factors provides immense value for understanding disease etiology in light of genomic variants. This immense value stems from the fact that a better characterization of mediators goes beyond statistical associations by providing a more complete picture of disease mechanisms via intermediary phenotypes, especially as they relate to trans-eGene expression [5, 6]. Furthermore, uncovering instances of cis-mediation also enables investigators to elucidate and describe regulatory networks with greater confidence, thereby providing a more systems-level understanding of psychiatric disorders. Within this framework, for instance, cis-eGenes that regulate the expression of many trans-eGenes would function as so-called “cis-hub” genes [7, 8], and these may form the basis of trans-eQTL hotspots within regulatory networks.

The Genotype-Tissue Expression (GTEx) project has identified both cis- and trans-eQTLs in multiple human tissues [9]. Nonetheless, the sample sizes available for brain subregions were fairly limited in GTEx, thereby providing only a limited number of identified trans-eQTLs in brain tissues. In our previously published work using PsychENCODE data [10], we only calculated and reported cis-eQTLs.

Here, we combined the large-scale data resources generated by the PsychENCODE Consortium, CommonMind (CMC), and GTEx to identify candidate trans-eQTLs with high confidence. We carried out a careful set of analyses on the features of these eQTLs and then compared them with those of cis-eQTLs. Furthermore, our analyses enabled us to identify trans-eQTLs hotspots. By both conducting statistical mediation analysis and integrating data on inter-chromosomal contacts, we evaluated two potential mechanisms by which variants may influence trans-eGene expression. Upon integrating our results with schizophrenia (SCZ) GWAS data, we demonstrate that these candidate trans-eQTLs might be pivotal for providing novel insights into disease mechanisms.

## Methods

### QTL analysis

We used the standard pipelines from ENCODE, GTEx, and other large consortia to uniformly process raw sequencing data from PsychENCODE [11] (1387 samples including RNA-seq and genotype data, see Availability of data and materials), as well as to identify functional genomic elements, such as brain enhancers, expressed genes, and eQTLs. We also processed other data types, such as Hi-C and single-cell data [10]. We used the imputed genotype and filtered expression data from our previous paper [10]. We harmonized the genotype and RNA-seq data from PsychENCODE. We used the Michigan imputation server [12] to impute the genotype data to the Haplotype Reference Consortium Panel [13]. We followed the GTEx pipeline for identifying all candidate trans-eQTLs to ensure maximal compatibility between our results and previous cis-eQTL results published by us and others. We found that lowly-expressed genes that were not detected in any samples can be falsely associated with SNPs due to the statistical fluctuation introduced by inverse quantile normalization. Therefore, after the gene filtering process in our previous paper, genes that were not detected in any of the 1387 samples were also removed from the analysis, leaving 12,278 genes. We used the QTLtools software package for candidate trans-eQTL identification [14]. Following the normalization scheme used by GTEx [9], the gene expression matrix was first normalized using quantile normalization, followed by inverse quantile normalization to map to a standard normal distribution (and to remove outliers). We used 50 probabilistic estimation of expression residuals (PEER) factors, 3 genotype principal components, gender, and the respective study as covariates in our calculations to identify cis-eQTLs (Given our much larger sample size, we used considerably more PEER factors than GTEx). The 50 PEER factors performed well in terms of capturing information on inter-sample differences in cell type abundances (Additional file 6: Fig. S1-5). For candidate trans-eQTLs, we calculated the associations between gene expression and variants greater than the 5 Mb window of each gene’s TSS (both upstream and downstream). These calculations were performed using genotype and gene expression data from 1387 individuals (associations between a total of 12k genes and 5,312,508 variants were tested for potential QTLs).

We performed multiple testing correction on nominal *P*-values by limiting pooled FDR values to less than 0.25 (Additional file 1) and 0.05 (Additional file 2) to generate two different lists of trans-eQTLs [15, 16]. We note that the list obtained using a pooled FDR threshold of 0.05 offers very limited power for downstream analyses. Therefore, with the goal of identifying as many trans-regulatory relationships as possible while simultaneously limiting the occurrence of false positives, we used the list with an FDR threshold of 0.25 for our analyses throughout our study. However, given that this FDR threshold still allows for more false positives than many studies involving QTLs, we have adopted the term “candidate trans-eQTLs” to refer to this list, wherever applicable. We identified ~77k candidate trans-eQTLs involving ~10K eGenes (Additional file 1). We generated an eGene list using hierarchical gene-level FDR list [17] (Additional file 3). We also used this hierarchical multiple test correction via a permutation-based scheme [9], and provide the trans-eQTL list at FDR<0.25 (Additional file 4).

### Enrichment of genomic elements

Using SnpEff [18], we annotated SNPs of cis-eQTLs, candidate trans-eQTLs, cQTLs, and all SNPs used for QTL calculations to identify cases in which SNPs overlap with genomic elements. We then tested for the enrichment of the QTL SNPs in different genomic elements using Fisher’s exact test by using all SNPs used for QTL calculations as the background. We also calculated the ratio of the number of SNPs in each genomic element to the total number of input SNPs. We selected promoters, untranslated regions, exons, introns, downstream regions, TF binding sites, enhancers, brain enhancers, and ePromoter regions for enrichment and ratio calculations.

### Mediation analysis

Candidate trans-eQTLs were further pruned for LD (r2>0.6), resulting in 74,143 independent candidate trans-eQTLs. We overlapped candidate trans- and cis-eQTLs that are in LD with a given independent candidate trans-eQTL and ran a colocalization analysis using the default settings of coloc [19]. Candidate trans- and cis-eQTL pairs that survived colocalization analysis with a posterior probability (H4 PP, the posterior probability indicating that cis- and candidate trans-eQTLs share causal variants) of greater than 0.5 were selected for mediation analysis. We used the SNP dosage and gene expression for the trans- and cis-eGenes associated with the SNP as input for the mediation analysis. We used the mediation package in R for the mediation analysis [20].

### Characterization of candidate trans-eQTLs that have cis-mediators

We identified the trans-eGenes that overlap with cis-eGenes, trans-eGenes that do not overlap with cis-eGenes, and cis-eGenes that are mediators for trans-eGenes. We then compared the effect sizes of eGenes in these three categories using the Kolmogorov–Smirnov test.

We hypothesize that cis-eQTLs may exert their effects on trans-eGenes via cis-eGenes that act as mediators. In this scenario, trans- and cis-eGene pairs that survive mediation analysis (mediation pairs) would need to be co-regulated. We therefore leveraged individual-level normalized expression data from Wang et al. [10] and measured expression correlation between cis- and trans-eGenes across 1,813 individuals. Because cis-mediators may act as both activators and repressors, we calculated absolute Pearson correlation coefficients associated with cis- and trans-eGene expression. Expression correlation coefficients of mediation pairs (mediation FDR<0.05) were then compared against expression correlations of random gene pairs (termed “random pairs”) and colocalized, but not mediated cis- and trans-eGene pairs (termed “coloc pairs”). To generate random pairs, pairs of genes were randomly selected after matching for the expression level with cis- and trans-eGene pairs. Coloc pairs were identified as cis- and trans-eGene pairs that are colocalized (H4 PP>0.5) but do not show evidence of mediation (mediation P>0.1).

We also surveyed the function of cis-mediators via gene ontology (GO) analysis using gProfiler [21]. We used all cis-eGenes as a background gene list. GO terms that survived multiple testing correction with a g:SCS threshold less than 0.05 were selected.

### Interchromosomal interactions

SNPs may affect genes located in another chromosome via interchromosomal interactions. We quantified interchromosomal interactions between trans-eSNPs and trans-eGenes (candidate trans-eQTL association FDR<0.25) using normalized chromatin contact matrices of the adult DLPFC [10] at a 100-kb resolution. Contact frequencies between trans-eSNPs and trans-eGenes were compared against contact frequencies between randomly selected 100-kb bins with matching chromosomes. Since interchromosomal contact frequencies are often zero, we only retained and compared non-zero interaction frequencies between candidate trans-eQTLs and random pairs.

### Trans-eQTL hotspots

Trans-eSNPs associated with three or more trans-eGenes were classified as trans-eQTL candidate hotspots. Among 74,143 independent candidate trans-eQTLs, 382 variants were identified as trans-eQTL hotspots. With the hypothesis that trans-eGenes associated with a given hotspot share a common trans-regulator, we evaluated whether trans-eGenes associated with hotspots may be more co-regulated than expected by chance. To this end, we calculated the mean absolute Pearson correlation coefficient among trans-eGenes associated with a given trans-eQTL hotspot using the individual-level normalized expression data from Wang et al. [10]. Mean absolute expression correlation coefficients were compared between trans-eQTL hotspots and randomly selected expression-matched gene sets.

### SCZ GWAS vs. candidate trans-eQTL colocalization

Because the number of candidate trans-eQTLs is much smaller than cis-eQTLs, we relaxed the thresholds for candidate trans-eQTLs in our colocalization analyses and retained only candidate trans-eQTLs with p<1e-5. We overlapped candidate trans-eQTLs (here, candidate trans-eQTLs with p<1e-5 were retained) with SCZ GWS loci (defined on the basis of LD [r2>0.6] with the index SNP, see Availability of data and materials) using the intersect function of bedtools. We then performed colocalization analysis between SCZ GWAS and candidate trans-eQTLs using the default setting of coloc [19]. Candidate trans-eQTLs that colocalized with SCZ GWAS at a threshold of H4 PP>0.6 were selected to identify trans-eGenes associated with SCZ (we refer to these as SCZ trans-eGenes).

To draw direct comparisons with SCZ trans-eGenes, SCZ cis-eGenes were defined using the same colocalization posterior probability as we had used for SCZ trans-eGenes (H4 PP>0.6). We filtered previously defined SCZ risk genes [10] using a threshold of H4 PP>0.6.

### Characterization of SCZ trans-eGenes

Genes with an excess of rare de novo LoF variation in SCZ were obtained from the SCHEMA browser [22]. We overlapped 32 genes that showed significant association with SCZ (at an FDR<0.05) [23] with SCZ trans-eGenes. We performed Fisher’s exact test between genes with SCZ-associated rare variation and SCZ trans-eGenes. Protein-coding genes were used as a background list in performing Fisher’s exact test.

To interrogate potential dysregulation of SCZ-trans/cis-eGenes in SCZ-affected individuals, we intersected these eGenes with genes that were differentially expressed in postmortem brain samples with SCZ (SCZ-DEGs) or co-expression modules associated with SCZ (SCZ-modules) [24]. SCZ-DEGs were selected based on FDR<0.05, regardless of whether the genes were upregulated or downregulated in SCZ. We ran Fisher’s exact test between SCZ-trans/cis-eGenes and SCZ-DEGs/SCZ-modules with the background list defined as genes that had been expressed in the work by Gandal et al. [24]. Because Gandal et al. [24] defined 34 co-expression modules (20 of which are associated with SCZ), P-values for over-representation analysis between SCZ-trans/cis-eGenes and SCZ-modules were corrected for multiple testing.

Network connectivity (kME values) was also obtained from Gandal et al. [24]. Each gene was assigned to a co-expression module via weighted gene co-expression network analysis [25, 26]. We quantified the kME value of a given gene in a co-expression module to which the gene belonged. We then compared the kME values of SCZ trans-eGenes with the kME values of SCZ cis-eGenes or all genes.

Next, we interrogated cellular expression profiles of SCZ-trans/cis-eGenes using single-cell RNA-seq data [10], as described previously [27]. We scaled expression profiles of each cell and calculated the average expression of SCZ-trans/cis-eGenes in a given cell. This cell-level expression value of a given gene was then aggregated based on cell type (neurons, astrocytes, microglia, endothelial cells, oligodendrocytes, and oligodendrocyte precursor cells) or neuronal subtype (excitatory and inhibitory subtypes).

### Trans-regulatory network linking variants and regulatory elements to genes

To analyze how eQTLs affect gene regulatory networks, we combined candidate trans-eQTLs with gene regulatory networks from four brain cell types: neurons (excitatory and inhibitory) and glial cells (microglia and oligodendrocytes). We mapped both cis-eQTLs and candidate trans-eQTLs onto the gene regulatory network, which may provide information about how eQTLs break certain TF binding sites and thereby result in changes to gene regulatory networks.

For the cis-network, we used all genes in eQTL pairs (SNPs and genes) as target genes (TGs). We filtered network edges by overlapping SNPs with binding sites on enhancers or promoters. Using motifbreakR [28], we then evaluated whether these SNPs break the corresponding TFs, which works with position probability matrices to interrogate SNPs for their potential effects on TF binding. We filtered the edges, thereby leaving only those edges that have SNPs with their target genes. The final results provided us with a dataset that contains each edge representing SNPs to TFs to enhancer/promoter to TGs linkages. We can visualize the results by plotting a genomic region surrounding certain SNPs, as well as potentially disrupted motifs.

Generating the trans-network entailed a similar procedure. However, for candidate trans-eQTL pairs, the SNPs may not be on the same chromosome as their target genes. When overlapping SNPs on binding sites, this introduces many challenges. Therefore, we used mediator-eQTL data instead of candidate trans-eQTL data when filtering out edges. After we carried out the same analysis as that detailed above (i.e., for the cis-network), we mapped mediators back to candidate trans-eQTLs (along with their target genes) to generate the results associated with the trans-network.

As a result, we built a mediator-candidate trans-cis-QTL gene regulatory network using mediators (TFs) and trans-network (TGs) cis-network results. This regulatory network links SNPs to mediators to the trans-genes that are regulated by them. Next, we utilized two types of predicted cell-type gene regulatory networks from scGRNom [29] for the major brain cell types: excitatory neurons (Ex1, Ex2, Ex3e, Ex4, Ex5, Ex6a, Ex6b, Ex8, and Ex9), inhibitory neurons (In1a, In1b, In1c, In3, In4a, In4b, In6a, In6b, In7, and In8), microglia, and oligodendrocytes. The first predicted gene regulatory network type corresponds to cell-type open chromatin regions from single-cell ATAC-seq data. The second predicted gene regulatory network type corresponds to a filtered network with the top 10% TFs that have absolute coefficients for a given target gene regardless of whether the region is characterized by cell-type-specific open chromatin. We then overlaid these TF-TG edges in both versions of cell-type gene regulatory networks with those in our mediator-candidate trans-cis-eQTL network to determine cell-type-specific TF-TG relationships detected by our mediator-candidate trans-cis-eQTL network.

## Results

### Identification of candidate trans-eQTLs in the human brain

Trans-eQTLs are especially difficult to identify in cohorts of limited sample sizes. To overcome this challenge, we worked with a large number of samples (*N*=1387, which includes the PsychENCODE brain resource, CMC, and GTEx brain samples), thereby more readily enabling us to identify candidate trans-eQTLs. We investigated the extent to which the resulting candidate trans-eQTLs might reveal potential mechanisms of distal regulatory linkages across chromosomes. We tested associations between 12,245 highly expressed genes and autosomal variants on a genome-wide scale (see Methods for processing and filtering criteria). We used the same covariates as those used for identifying cis-eQTLs in our previously published work [10]. We removed genes with poor mappability and variants located in repetitive regions. Furthermore, to minimize false positives, we filtered out candidate trans-eQTLs between pairs of genomic loci with evidence of RNA-sequencing (RNA-seq) read cross-mapping [30]. We calculated false discovery rate (FDR) values from the linkage-disequilibrium (LD)-pruned list of candidate trans-eQTLs to determine significance. We employed the following three ways of performing multiple test correction to call trans-eQTLs, and we provide the lists called with each of these methods as Additional files.

**Fig. 1:**
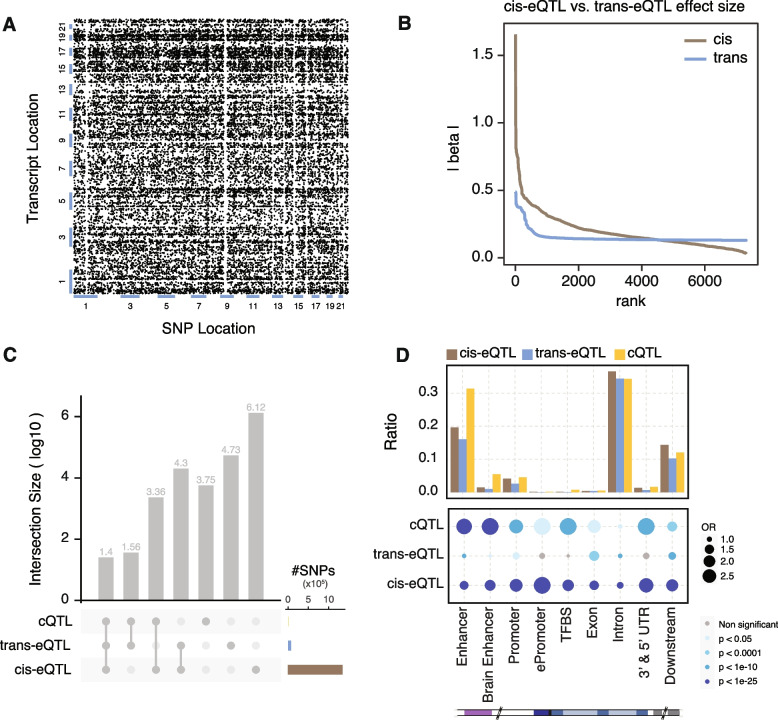
Characterization of candidate trans-eQTLs. **A** Genetic map for candidate trans-eQTLs. **B** Comparisons of the effect sizes between cis-eQTLs and candidate trans-eQTLs. **C** Frequencies with which SNPs are shared among various combinations of the 3 QTL types (cis-eQTLs, candidate trans-eQTLs, and cQTLs). **D** Enrichment statistics and variant proportions for the frequencies with which cis-eQTLs, candidate trans-eQTLs, and cQTLs lie within various types of genomic elements

1. Pooled genome-level FDR correction method per GTEx 2017 [17]. At an FDR threshold of 0.25, we detected 77,156 candidate trans-eSNPs from ~5.3M total SNPs tested in locations ≥5 Mb from the gene transcription start site (TSS), comprising 17,899 independent SNPs after LD pruning (Fig. 1A). We term this list (at FDR<0.25) “candidate trans-eQTLs” and provide it in Additional file 1. Separately, we compiled alternate lists at different confidence thresholds. In particular, we identified 7642 candidate trans-eQTLs involving 580 eGenes at an FDR threshold of 0.05; this list is available in Additional file 2.
2. Hierarchical gene-level FDR correction per GTEx 2017 [17]. This method generates an eGene list. We provide this list (at a gene-level FDR<0.25) in Additional file 3.
3. Permutation-based hierarchical gene-level FDR per GTEx 2020 [9]. We used this hierarchical multiple test correction via a permutation-based scheme and provide the list (at FDR<0.25) in Additional file 4.

We performed an internal replication validation for the candidate trans-eQTLs by splitting the samples randomly to two folds with no overlapping samples. Two sets of candidate trans-eQTLs were then called after this splitting: one set was called using only the samples in fold 1, and the other set was called using only the samples in fold 2 (i.e., the trans-eQTLs were calculated within each fold separately). We then quantified the similarities between these 2 sets of candidate trans-eQTLs by measuring the overlap of trans-eQTLs from each of the two folds. We found that the overlap of the trans-eQTLs generated by these two folds is around 80% at an FDR threshold of 0.05.

Relative to previously published studies, we identified substantially more candidate trans-eQTLs and associated eGenes in the human brain by leveraging integrated data resources. We used the candidate trans-eQTL list with FDR<0.25 for the analyses discussed in this study (i.e., those provided in Additional file 1).

We next characterized the genomic features of candidate trans-eQTLs and compared them with other types of QTLs in order to investigate associations between genomic elements and QTLs (Fig. 1B–D). In agreement with previous findings, we found that the magnitude of the effect sizes associated with cis-eQTLs are larger than that those of candidate trans-eQTLs (Fig. 1B). Candidate trans-eQTLs overlapped more frequently with cis-eQTLs than with chromatin QTLs (cQTLs) [10], which is also largely expected. The low overlap of cQTLs and candidate trans-eQTLs may be a consequence of the overall low number of cQTLs. We found 20,175 eSNPs that are shared between cis- and candidate trans-eQTLs, whereas only 61 SNPs were shared between candidate trans-eQTLs and cQTLs. As shown in Fig. 1C, we found 25 SNPs that are shared among all three QTL types (i.e., cis-eQTLs, candidate trans-eQTLs, and cQTLs). With respect to genomic elements, we found that candidate trans-eQTLs tend to exhibit lower enrichment in most elements relative to cis-eQTLs and cQTLs; however, candidate trans-eQTLs are most enriched within exons (Fig. 1D). The pattern of variant proportion on different genomic regions for candidate trans-eQTLs is similar to that for cis-eQTLs but not cQTLs. We also calculated the enrichment of the QTLs in previously published brain enhancer lists [31] and found the enrichment patterns to be similar across these enhancers (Additional file 6: Fig. S6A).

### Potential mechanisms of candidate trans-eQTLs

**Fig. 2:**
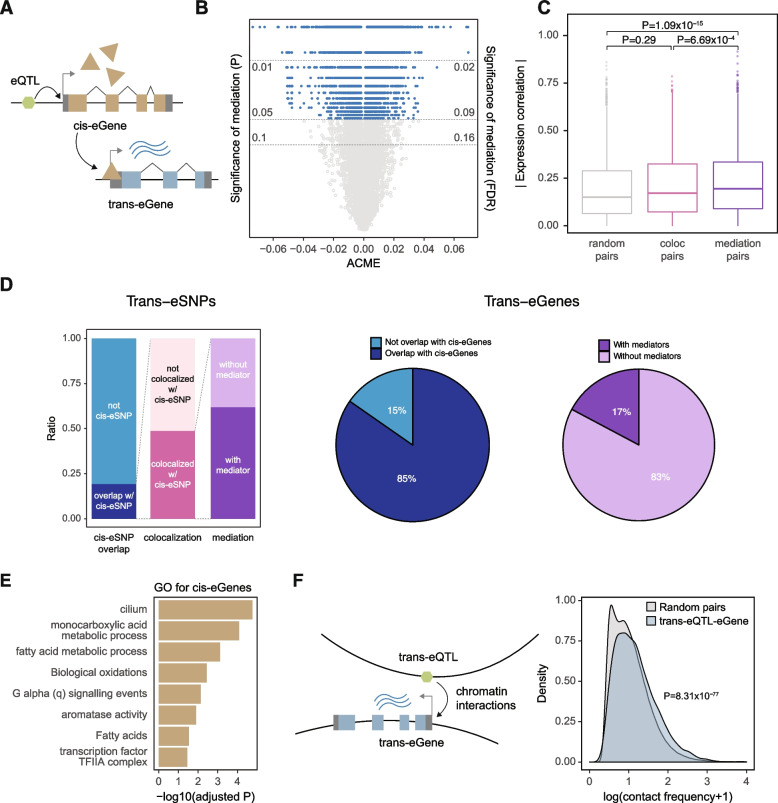
Cis-mediation and inter-chromosomal interactions explain candidate trans-eQTL associations. **A** A candidate trans-eQTL may overlap with a cis-eQTL that regulates a proximal gene (cis-eGene), which may in turn regulate a distal gene (trans-eGene). **B** Roughly 62% of candidate trans-eQTLs colocalized with cis-eQTLs exhibit mediation effects. The number of candidate trans-eQTLs with positive mediation effect sizes is almost the same as those that are negative. **C** Cis- and trans-eGene pairs with evidence of cis-mediation display significant co-expression compared to both random pairs and those pairs that exhibit colocalization but not mediation. **D** Relative abundances of candidate trans-eQTLs that overlap with cis-eQTLs (left), colocalize with cis-eQTLs (middle), and exhibit mediation effects (right). Roughly 85% of trans-eGenes are also cis-eGenes, and ~17% of trans-eGenes have cis-mediators. **E.** Biological pathways associated with cis-eGenes that mediate candidate trans-eQTL associations are enriched for metabolic processes and transcriptional regulation. **F** Candidate trans-eQTL and eGene pairs tend to exhibit inter-chromosomal interactions more frequently than do random pairs. ACME, average causal mediation effects

In one potential mechanism, specific variants exert trans-regulatory effects by influencing a nearby regulatory factor (such as a TF), which in turn regulates distal genes in a trans fashion [3]. In this scenario, trans-eQTLs may also act as cis-eQTLs for nearby genes that have regulatory impacts on trans-eGenes (Fig. 2A). Indeed, we found that 19.33% of LD-pruned candidate trans-eQTLs display cis-eQTL signals (Fig. 2D). Because simple genomic coordinate-level overlaps between cis- and candidate trans-eQTLs may detect spurious associations due to LD, we performed a colocalization assay to identify cis- and candidate trans-eQTL pairs that harbor shared causal variants. In total, we detected 1688 candidate trans-eQTLs (48.79% of the candidate trans-eQTLs that overlap with cis-eQTLs) that have shared causal variants with cis-eQTLs. We further interrogated the potential causal effects of cis-eQTLs on trans-eQTL associations via mediation analysis (Fig. 2B, Additional file 5). As part of this analysis, we found that 62.13% of the candidate trans-eQTLs that colocalize with cis-eQTLs can be explained by cis-mediators (*p*<0.05). Among the candidate trans-eQTLs that can be explained by cis-mediators, there is roughly an even split of cis-mediators with positive and negative mediation effects, suggesting that trans-regulators can either be activators or repressors with roughly equal probability (Fig. 2B). As generally expected, we also observed that larger mediation coefficients tend to have greater statistical significance. We found that 85% of trans-eGenes are also cis-eGenes, indicating that gene expression is regulated by both cis- and trans-acting variants. Roughly 20% of trans-eGenes had cis-eGenes as mediators (Fig. 2D).

By definition, because cis-mediation implies that variants influence trans-eGene expression via cis-eGenes, we hypothesized that cis- and trans-eGene pairs with evidence of cis-mediation are co-regulated. We evaluated this hypothesis by first grouping cis- and trans-eGene pairs into those that exhibit evidence of cis-mediation (which we term “mediation pairs”) and those with evidence of colocalization but not mediation (which we term “colocalization pairs”). When comparing these groups, we found that mediation pairs showed greater expression correlation than colocalization pairs or expression-level matched random pairs (Fig. 2C; see the “Methods” section), thereby providing additional evidence for cis-mediation. We also observed that trans-eSNPs are enriched on exons (Fig. 1D). Therefore, one possible mediating mechanism may indeed be non-synonymous coding variation in the mediating genes (Additional file 6: Fig. S6B).

We next investigated the properties of cis-eGenes that were found to mediate trans-regulatory effects. In addition to being enriched for transcriptional regulators (e.g., members of TF complexes), cis-eGenes were also enriched for other biological processes (e.g., metabolic processes, Fig. 2E). These results indicate that variant effects on distal gene regulation are not solely dependent on TF activity. Instead, variants that are associated with metabolism may exert broad systems-level effects on cellular function, which can then lead to changes in distal gene expression.

Another potential explanation for trans-eQTLs may lie in inter-chromosomal interactions. Previous studies have shown that inter-chromosomal interactions can bring multiple genes from different chromosomes into close physical proximity, thereby more easily enabling these genes to be co-regulated [32]. We therefore investigated the extent by which trans-eSNPs may regulate trans-eGenes located in different chromosomes via inter-chromosomal interactions. We observed that candidate trans-eSNP-eGene pairs display increased chromatin contact frequency compared with random inter-chromosomal contacts (Fig. 2F). Hence, in addition to cis-mediation, trans-eQTL associations can be partly driven by features of chromosomal conformation.

### Trans-eQTL hotspots

**Fig. 3:**
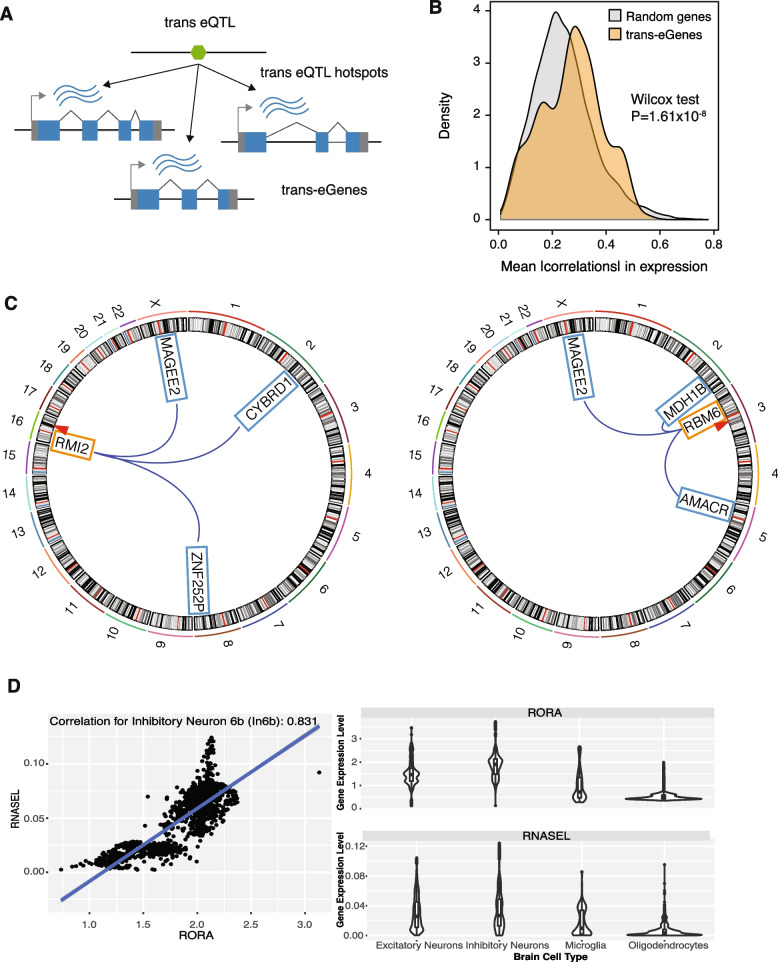
Trans-eQTL hotspots. **A** Trans-eQTL hotspots represent trans-eSNPs that exhibit associations with at least three trans-eGenes. **B** Trans-eGenes associated with a shared trans-eSNP tend to be co-regulated. **C** Examples of trans-eQTL hotspots. The cis-eGenes are shown in orange boxes, and their respective trans-eGenes are shown in blue boxes. The red triangles are used to schematically designate the proximal relationship between the cis-eSNP and cis-eGene. **D** Cell-type-specific TF-target gene relationships detected by our mediator-candidate-trans-cis-QTL network

Trans-regulators exert broad impacts on regulatory landscapes by affecting many downstream targets. Given the trans-regulatory properties of trans-eQTLs, some trans-eSNPs may affect multiple genes, thereby forming “trans-eQTL hotspots.” We defined such trans-eQTL hotspots as candidate trans-eSNPs that affect three or more genes (Fig. 3A). In total, we detected 382 trans-eQTL hotspots. Because trans-eGenes for a given trans-eQTL hotspot are regulated by the same SNP, we expect that they might generally be co-regulated. Indeed, we found that trans-eGenes grouped by hotspots are significantly more co-regulated compared to expression-level-matched random controls (Fig. 3B).

One of the identified trans-eQTL hotspots consists of three trans-eGenes (*MAGEE2*, *CYBRD1*, *ZNF252*), which are distributed across the genome and are regulated by a trans-eSNP (chr16:11394372; Fig. 3C). Notably, this trans-eSNP was a cis-eQTL for *RMI2*, a gene associated with genome instability and Bloom syndrome [33]. In another example, the cis-eSNP for *RBM6* (chr3:50257020), an RNA-binding protein, was associated with three trans-eGenes (*MAGEE2*, *MDH1B*, *AMACR*, Fig. 3C). Collectively, these results suggest that multiple biological processes (such as genome instability and RNA processing) may exert broad impacts on gene regulation via trans-regulatory mechanisms.

### Cell-type gene regulatory effects of candidate trans-eQTLs and mediators

Because candidate trans-eQTLs were defined from the brain homogenate and lack cell-type specificity, we evaluated cell-type-specific trans-regulatory effects from our mediators to trans-genes. For instance, our mediation analysis indicated that retinoic acid-related orphan receptor alpha (RORA, a nuclear receptor TF) is a mediator of the trans-eGene *RNASEL,* which encodes mammalian endoribonuclease. Based on single-cell multi-omics data, we found that RORA regulates the gene *RNASEL* specifically in neuronal cell types [29]. As shown in Fig. 3D, cellular expression levels of *RORA* and *RNASEL* show high Pearson correlation coefficients in several neuronal cell types, especially in inhibitory types, such as In6b (*r* = 0.831), In8 (*r* = 0.790), Ex9 (*r* = 0.746), and In6a (*r* = 0.742), compared to glial cell types such as microglia (*r* = 0.6436) and oligodendrocytes (*r* = 0.492).

Both of these genes have been implicated in disorders of the brain. For example, the overactivation of RNASEL may contribute to neurodevelopmental and inflammatory genetic disorders such as Aicardi–Goutières syndrome [34]. The expression of RNASEL may increase as a result of NMDA receptor activation in cortical neurons by glutamate, thus resulting in degradation of RNA molecules. Furthermore, degradation of mitochondrial RNA by RNASEL may contribute to neuronal death overall [35].

Reduced levels of the nuclear receptor TF RORA have been observed in the prefrontal cortex and cerebellar neurons of individuals with autism spectrum disorder (ASD) [36], and retinoic acid signaling pathways have been reported to be disrupted in ASD-afflicted individuals [37]. The decreased expression of RORA impacts the regulation of its target genes in ASD-afflicted individuals (several of which are ASD-relevant genes) and is associated with the pathobiology of ASD, such as with decreases in neuronal differentiation and survival, poorer synaptic transmission and neuroplasticity, diminished cognition and spatial learning, memory impairment, and disrupted development of the cortex and cerebellum [36]. Furthermore, studies have found that RORA is involved in the differentiation of Purkinje cells, development of the cerebellum region, protection of neurons against oxidative stress, circadian clock regulation, and suppression of inflammatory processes [36].

### Candidate trans-eQTLs identify novel disease mechanisms

It has been proposed that trans-eQTLs explain 60–90% of gene expression heritability [38, 39]. However, functional annotation of GWAS variants largely relies on the use of cis-eQTLs, which may miss key biological underpinnings of human traits and diseases. Trans-eQTLs may provide novel insights into the biological mechanisms underlying psychiatric illnesses. Therefore, we performed colocalization analysis [19] between SCZ GWAS [40] and candidate trans-eQTLs to unveil previously uncharacterized SCZ-associated biological pathways driven by trans-regulatory mechanisms (“Methods”). We then compared these results with the colocalization results between SCZ GWAS and cis-eQTLs. We found that some loci only colocalized with cis- or candidate trans-eQTLs, although several colocalized with both.

**Fig. 4:**
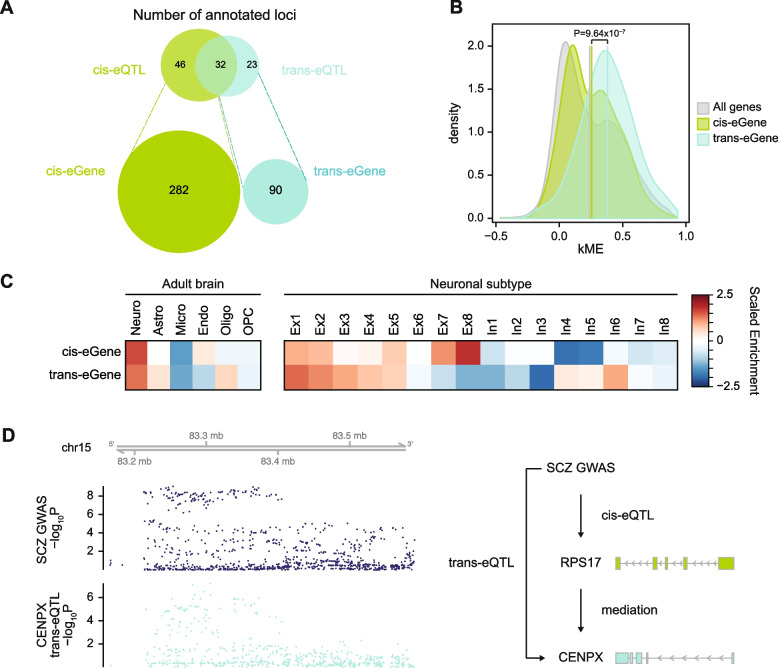
Candidate trans-eQTLs identify novel SCZ risk genes and biological pathways. **A** The numbers of SCZ GWS loci annotated by cis- and candidate trans-eQTLs. SCZ cis-eGenes do not overlap with SCZ trans-eGenes. **B** Trans-eGenes that colocalize with SCZ risk genes exhibit greater network centrality. **C** Cellular expression profiles of SCZ-associated cis- and trans-eGenes. **D** A SCZ GWS locus colocalizes with candidate trans-eQTLs for *CENPX* (left). This GWS locus is a cis-eQTL for *RPS17*, a cis-mediator of *CENPX* (right)

In total, we found that candidate trans-eQTLs could explain 55 out of 142 SCZ-associated genome-wide significant (GWS) loci (Fig. 4A). In contrast, cis-eQTLs annotated 78 GWS loci (Fig. 4A). 32 GWS loci colocalized with both cis- and candidate trans-eQTLs, suggesting that a subset of SCZ loci may exert their effects via multiple regulatory mechanisms. Furthermore, 23 GWS loci colocalized only with candidate trans-eQTLs but not with cis-eQTLs, suggesting that trans-eQTLs may provide regulatory mechanisms for previously unexplained loci.

A colocalization analysis resulted in 90 and 282 SCZ-associated trans- and cis-eGenes, respectively; we refer to these as SCZ-(trans/cis)-eGenes (Fig. 4A). As expected, none of the SCZ-cis- and SCZ-trans-eGenes overlapped, demonstrating that candidate trans-eQTLs can pinpoint distinct SCZ-associated genes and biological pathways. In particular, SCZ trans-eGenes were found to be enriched for JUN kinase activity (FDR=0.0056), a signaling pathway involved in neuronal apoptosis, neurite outgrowth, and dendrite arborization [41].

Notably, two of the 90 SCZ trans-eGenes (*AKAP11*, *SETD1A*) also harbor SCZ-associated rare variants (de novo loss-of-function [LoF] variation) [23] (Fisher’s exact test, *P*=9.6×10^−3^, odds ratio [OR]=14.62, 95% confidence interval [CI]=1.67–59.06; see Additional file 6: Fig. S7 for colocalization between SETD1A candidate trans-eQTLs and SCZ GWAS). This contrasts with the 282 SCZ cis-eGenes, none of which overlapped with the genes that harbor SCZ-associated rare de novo LoF variants. This result lends support to the omnigenic hypothesis [38], which posits that genes can be divided into core genes that directly affect the disease-related biological processes and peripheral genes that regulate these core genes. Core genes are likely to be affected by rare variants with large effect sizes, suggesting that these two SCZ trans-eGenes are likely core genes. In addition, the hypothesis suggests that core genes are likely to be targeted by trans-regulatory mechanisms, which is consistent with these two genes being trans-eGenes of SCZ rare variants that are mediated by peripheral genes.

To provide additional support for the omnigenic hypothesis, we explored the network properties of SCZ trans-eGenes. Core genes are thought to be enriched for network hubs [42]. Therefore, we measured module membership (kME; a measure of network centrality) of SCZ-eGenes in brain co-expression networks constructed from the same samples [24]. SCZ trans-eGenes showed significantly higher kME values than did cis-eGenes or brain-expressed genes in general (Fig. 4B, two-sample Wilcoxon test: cis vs. trans, *P*=9.64×10^−7^; all vs. trans, *P*=2.87×10^−9^; all vs. cis, *P*=9.9×10^−2^). These results demonstrate that SCZ trans-eGenes are characterized by greater network connectivity.

We found that a significant portion of SCZ-associated trans- and cis-eGenes were differentially regulated in brain tissue of SCZ-affected individuals [42] (Fisher’s exact test: trans, *P*=9.4×10^−3^, OR=1.87, 95% CI=1.14–2.98; cis, *P*=3.5×10^−2^, OR=1.38, 95% CI=1.01–1.85). However, they were enriched in different SCZ-associated co-expression networks. SCZ trans-eGenes showed selective enrichment in the SCZ-associated gene module 7 (geneM7, beta=0.0033, FDR=0.011), a neuronal module involved in synaptic vesicle formation that exhibits elevated expression signatures in SCZ (Fisher’s exact test: P=8.02×10^−5^, FDR=0.0014, OR=5.52, 95% CI=2.42–11.10). In contrast, SCZ cis-eGenes were only nominally enriched in gene module 8 (geneM8, beta=-0.0030, FDR=0.017), a neuronal module downregulated in SCZ (Fisher’s exact test: *P*=1.8×10^−2^, FDR=0.53, OR=2.31, 95% CI=1.13–4.24). Therefore, SCZ-associated trans- and cis-eGenes may account for different expression signatures of SCZ.

Cell-type expression profiles of SCZ-eGenes illustrated potential cell types that contribute to distinct expression features (Fig. 4C). Both SCZ-associated trans- and cis-eGenes were highly expressed in neurons, which is consistent with previous findings that neurons are the primary cell type underlying SCZ etiology [27, 43–46]. However, SCZ cis-eGenes exhibited relatively higher expression in lower-layer neurons (Ex7-8), while SCZ trans-eGenes showed upper-to-lower-layer gradient expression. Furthermore, while SCZ cis-eGenes were relatively depleted in inhibitory neurons, SCZ trans-eGenes were highly expressed in parvalbumin-expressing GABAergic interneurons (In6). One example of a SCZ-trans-eGene is *CENPX*, which is associated with kinetochore assembly and DNA damage repair [47]. SCZ GWAS colocalized with candidate trans-eQTLs for *CENPX*, which was mediated by cis-eQTLs for *RPS17*, a gene that encodes a ribosomal protein (Fig. 4D). Ribosomal proteins can affect translation efficiency and mRNA stability. This result suggests that an SCZ GWAS SNP may affect the nearby gene *RPS17*, which potentially alters the mRNA stability and in turn regulates *CENPX*, a gene located in a different chromosome. Together, these results demonstrate how the integration of GWAS variants, cis-eQTLs, and candidate trans-eQTLs can enhance our mechanistic understanding of disease etiology.

## Discussion

Coupled with increased multiple testing burdens, the smaller effect sizes of trans-eQTLs make their identification considerably more challenging than those of cis-eQTLs. Indeed, we found that candidate trans-eQTLs exhibit smaller effect sizes compared with cis-eQTLs. Furthermore, candidate trans-eQTLs were less enriched in regulatory elements compared with cis-eQTLs, suggesting that trans-regulatory architectures may differ from those associated with cis-regulation.

Despite these differences, we found that a significant portion of candidate trans-eQTLs overlapped with cis-eQTLs, suggesting that trans-acting variants may often exert their effects via cis-mediators. We found that trans-eGenes with evidence of cis-mediators displayed larger effect sizes and co-expression signatures with cis-mediators. Intriguingly, cis-mediators were involved in fatty acid metabolism, which contributes to ~20% of the brain’s energy reserves [48]. This result indicates that trans-regulatory mechanisms may involve various biological processes that influence fundamental cellular function and psychiatric disorder progression.

We also found that, in addition to cis-mediation, candidate trans-eQTLs may result from inter-chromosomal interactions. Elucidating the mechanisms that underlie trans-eQTL associations will provide important insights into trans-regulatory networks.

We further deconvolved trans-regulatory networks into specific cell-type-specific trans-regulatory networks and investigated the potential effects of the identified mediators on trans-eGenes. These analyses uncovered a strong link between the regulation of *RORA* and *RNASEL*, both of which are associated with the immune system response and naive T cell states (they also harbor polymorphisms associated with elevated cancer risk and mortality). Beyond psychiatric disorders, additional disorders have been linked to *RORA* and *RNASEL*. For example, both genes have been shown to be associated with prostate cancer. In affected men, *RORA* is typically inactivated [49], and *RNASEL* is a candidate for the hereditary prostate cancer gene (*HPC1*) [50]. In fact, some mutations in the tumor-suppressor gene *RNASEL* have been found to lead to ribonuclease L dysfunction, inflammation, infection, and increased risk of prostate cancer, suggesting links between innate immunity and tumor suppression [51]. These findings can help provide greater insights into age-related changes, the roles of common fragile sites and genomic instability, the roles of the adaptive immune response (especially of T cells), and the potential roles of the central nervous system in the onset and progression of one of the world’s leading cancer types [52].

Given the differences between cis- and trans-eQTLs, we hypothesized that trans-eQTLs might provide novel insights into the biology of psychiatric disorders. Motivated by this notion, we related candidate trans-eQTLs to SCZ GWAS variants to decipher trans-regulatory mechanisms that may contribute to SCZ etiology. The candidate trans-eQTLs led to the identification of novel SCZ risk genes, a subset of which could explain previously uncharacterized SCZ GWS loci. In total, candidate trans-eQTLs assigned 55 out of 142 SCZ GWS loci.

SCZ candidate trans-eGenes differed from SCZ cis-eGenes in multiple respects. For example, SCZ trans-eGenes included *SETD1A* and *AKAP11*, both of which are high-confidence SCZ risk genes that harbor rare de novo LoF variations [23]. In contrast, SCZ cis-eGenes did not overlap with any of the rare variation-targeted SCZ risk genes. *SETD1A* encodes a histone methyltransferase. Mice that carry a LoF mutation in *SETD1A* showed cognitive deficits, abnormal neuronal morphology, and transcriptional alterations, further implicating its role in SCZ etiology [53]. Moreover, we observed enhanced network centrality of SCZ trans-eGenes compared with SCZ cis-eGenes. This is consistent with our recent finding that hub genes are not enriched for SCZ heritability when cis-regulatory mechanisms are used to map genetic risk factors to genes [54]. These characteristics of SCZ trans-eGenes (e.g., overlap with rare variation and network centrality) correspond to the definition of the core genes from the omnigenic hypothesis [38] that are likely to be trans-regulated by rare variants, suggesting that trans-eQTLs may play crucial roles in understanding core biological principles of SCZ. While this observation warrants further investigation when a larger set of rare variant-harboring SCZ risk genes and/or a more comprehensive list of trans-eQTLs becomes available, the observed distinction between SCZ cis- and trans-eGenes highlights the importance of characterizing trans-regulatory mechanisms in SCZ pathogenesis.

Cellular expression profiles further substantiated distinct biological processes represented by SCZ-associated trans- and cis-eGenes. While SCZ cis-eGenes were enriched in lower-layer neurons, SCZ trans-eGenes were enriched in upper-layer neurons, suggesting that cis- and trans-regulatory mechanisms may influence distinct cortical circuitry. Moreover, SCZ trans-eGenes were enriched in parvalbumin-expressing interneurons, which have reported genetic and transcriptional associations with SCZ [55, 56].

To further demonstrate regulatory mechanisms of trans-eQTLs and their implications in psychiatric disorder etiology, we note that the trans-eQTLs identified in this study warrant experimental validation. Experimental biologists may apply genome editing to brain cell models, e.g., induced Pluripotent Stem Cells (iPSC) derived neurons, to directly evaluate trans-eQTLs and to measure the expression of trans-eGenes. However, it is of note that the adult brain tissue from which we have obtained candidate trans-eQTLs displays high levels of cellular heterogeneity, which makes it difficult to model using iPSC-derived cell lines. Building a cell-type specific eQTL resource from the brain tissue [57] will be critical to address cellular heterogeneity, while obtaining large sample sizes to discern trans-eQTLs will remain a challenge.

## Conclusions

In this work, we report one of the first systematic searches and detailed studies of genome-wide candidate trans-eQTLs in the dorsolateral prefrontal cortex (DLPFC). By leveraging the extensive resource built by the PsychENCODE consortium [10], we detected 77,304 candidate trans-eQTLs (at an FDR threshold of 0.25) and 382 trans-eQTL hotspots. We reasoned that trans-eQTLs may provide new avenues for investigating trans-regulatory mechanisms, thereby providing critical insights into the regulatory landscape in psychiatric disorders and human health. In conclusion, the transcriptional architecture of the human brain is orchestrated by both cis- and trans-regulatory variants, and trans-eQTLs can provide novel insights into human brain disease biology.

## Supplementary Information

**Additional file 1.** Candidate trans-eQTLs with pooled FDR<0.25 in DLPFC.

**Additional file 2.** trans-eQTLs with pooled FDR<0.05 in DLPFC.

**Additional file 3.** trans-eGenes with gene level FDR<0.25 in DLPFC.

**Additional file 4.** trans-eQTLs with permutation FDR<0.25 in DLPFC.

**Additional file 5.** Mediation analysis results for candidate trans-eQTLs.

**Additional file 6.** Supplementary figures and analysis of PEER factors to mitigate confounding effects of inter-sample cellular heterogeneity.

## Data Availability

The PsychENCODE RNA-seq data, genotype data, demographics data, and other genomic data analyzed in this study are accessible at the PsychENCODE knowledge portal (https://psychencode.synapse.org/Explore/Data and http://resource.psychencode.org/ )[10, 11, 24]. The GTEx data used for the trans-eQTL analysis in this study are accessible at dbGaP Study Accession: phs000424.v9.p2 (https://www.ncbi.nlm.nih.gov/projects/gap/cgi-bin/study.cgi?study_id=phs000424.v9.p2) [6, 9, 17]. The schizophrenia GWAS data used in this study is from the CLOZUK+PGC2 study (http://walters.psycm.cf.ac.uk/) [40].
