# Supplementary material for: Illuminating links between cis-regulators and trans-acting variants in the human prefrontal cortex
Source: Genome Med. 2022 Nov 24;14:133. doi: 10.1186/s13073-022-01133-8 (PMC9685876; doi:10.1186/s13073-022-01133-8)
Supplement: Supplementary file 6 — Additional file 6. Supplementary figures and analysis of PEER factors to mitigate confounding effects of inter-sample cellular heterogeneity. [file 13073_2022_1133_MOESM6_ESM.docx]

**PEER factors to mitigate confounding effects of inter-sample cellular heterogeneity**

Batch effects and other hidden covariates present considerable challenges in QTL analyses. In particular, inter-sample differences in cell-type compositions or relative fractions likely comprise important covariates for QTL identification and would thus constitute serious confounders if not properly controlled.

We used 50 PEER factors to account for hidden covariates in our analysis. Thus, to address the challenge of cellular heterogeneity as a potential confounder, we carried out two separate but complementary approaches to investigate the degree to which these PEER factors capture information on cell type heterogeneity. To the extent that such inter-sample differences in cell-type abundances are reflected in the PEER factors, we are largely able to control for these inter-sample differences through our use of PEER factors.

We found that the 50 PEER factors used in our QTL searches perform extremely well in terms of capturing information on cell type composition and heterogeneity. We first carried a multidimensional projection analysis to demonstrate this. As linearly independent vectors in a higher-dimensional space (with the space of this dimension being equal to the number of samples), these 50 PEER factors constitute a 50-dimensional subspace that is embedded within the higher-dimensional space.

These notions are schematized simply in Fig. S1. For ease of illustration, this figure shows only two **PEER_factor** vectors, which in turn define a 2-dimensional subspace embedded within the higher-dimensional space (again, for ease of illustration, this higher-dimensional space is only 3-dimensional, for ease of visualization). For a given cell type, the **Cell_fract** vector represents the cell fractions in all samples in our study (for example, **Cell_fract** is a vector that may represent the fraction of Astrocyte cells in all individuals). In the schematic shown in Fig. S1, the **Cell_fract** vector does not lie within the 2D PEER space (i.e., the subspace spanned by the PEER factors). This is because there exists no linear combination of the two PEER factors that perfectly reconstitutes the **Cell_fract** vector.


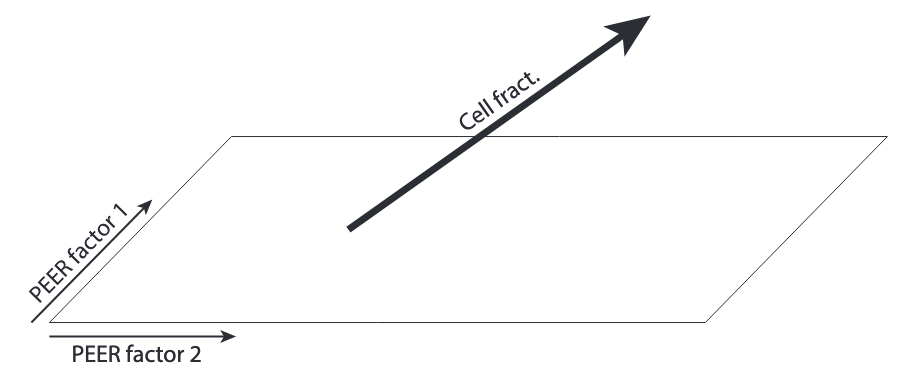


**Fig. S1.** Schematic visualization of how the cell fractions (for example, the fractions of Astrocyte cells) across individuals may be represented in a high-dimensional space, within which lies a lower-dimensional PEER subspace. Here, 2 PEER factors (i.e.,  **PEER_factor** vectors) define a 2D subspace that is embedded within a higher-dimensional 3D space. The **Cell_fract** vector also lies within the higher-dimensional space, but it may not lie within the 2D subspace defined by the **PEER_factor** vectors.

Our objective was thus to evaluate the degree to which we may approximate heterogeneity in cell fractions (i.e., a **Cell_fract** vector) using a linear combination of the 50 PEER factors. The components that make up the **Cell_fract** vector may be expressed as a linear combination of the 50 **PEER_factor** vectors as well as an **Error** vector:

**Cell_fract**  =  β_1_ **PEER_factor_1**  +  β_2_ **PEER_factor_2**  +  ...  +  β_50_ **PEER_factor_50**  +  **Error**

This is demonstrated schematically in Fig. S2., where we again show only 2 PEER factors for ease of visualization. Here, the specific linear combination of **PEER_factor** vectors that achieves the best approximation to the **Cell_fract** vector (in terms of having a minimum length associated with the **Error** vector) is obtained via an orthogonal projection of **Cell_fract** onto the PEER subspace.


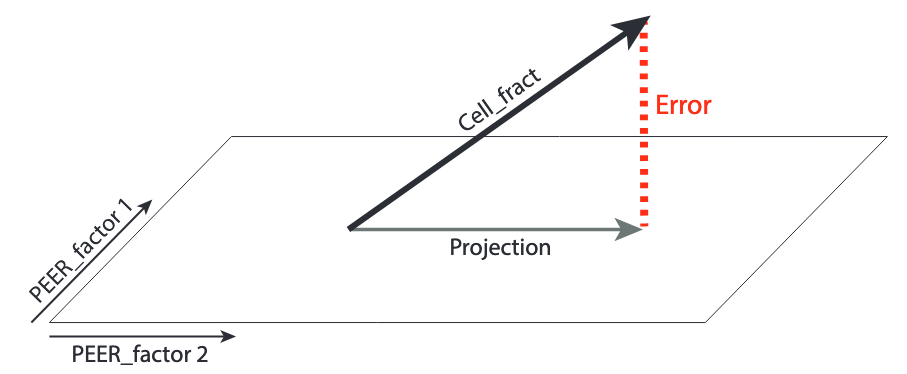


**Fig. S2.** An orthogonal projection of the **Cell_fract** vector onto the subspace spanned by the PEER factors provides the best possible approximation of the **Cell_fract** vector using a linear combination of the PEER factors. This approximation is given by the **Projection** vector. The difference between this **Projection** vector and the **Cell_fract** vector is given by the **Error** vector, the length of which quantifies the accuracy of the approximation.

The 50 **PEER_factor** vectors are stored in a matrix **P**, and the coefficients that make up the optimal linear combination of **PEER_factor** vectors are stored in a vector **β**:


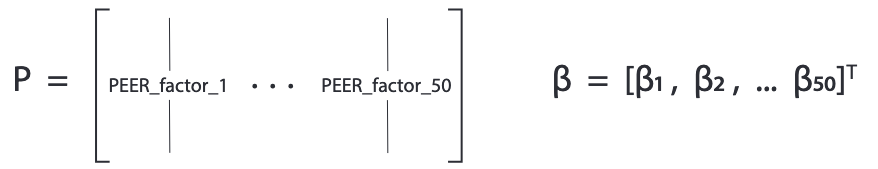


Thus, in matrix notation,

**Cell_fract**  =  **Pβ**  +  **Error**

The **Projection** vector is given by:

**Projection**  =  **P** [**P**^T^**P**]^-1^ **P**^T^ **Cell_fract**

By measuring the error term associated with a given projection, we can quantify the degree to which the PEER factors capture information on differences in cell type abundances across individuals for a given cell type. A large error (as in case I in Fig. S3) suggests that the PEER factors are effectively blind to differences in cell type fractions and PEER factors thus fail to account for inter-sample differences in cellular composition, whereas an error closer to zero (as in case II in Fig. S3) suggests that the PEER factors more accurately capture inter-sample differences in a given cell type’s relative abundances.

To measure the extent to which our set of 50 PEER factors constitute a basis set of vectors for approximating cellular fractions for a given cell type, we compared the length of our observed **Error** vector with an expected **Error** vector length under a null model in which the PEER factors were truly independent of (i.e., blind to) cell fractions. These null models were generated empirically using a simulation-based scheme in which we randomly permuted the values within the given **Cell_fract** vector 10,000 times and storing the associated errors. By randomly permuting the values in the **Cell_fract** vector in this way, we are effectively re-orienting the vector in random directions within the high-dimensional space while conserving the length of the **Cell_fract** vector. We then plotted the 10,000 corresponding **Error** vector lengths from these randomly-oriented Cell_fract vectors, and the results are given in Fig. S4. The p-values indicate the fraction of randomly-oriented (i.e., simulated) **Cell_fract** vectors that may be better approximated using the PEER factors than an observed **Cell_fract** vector.


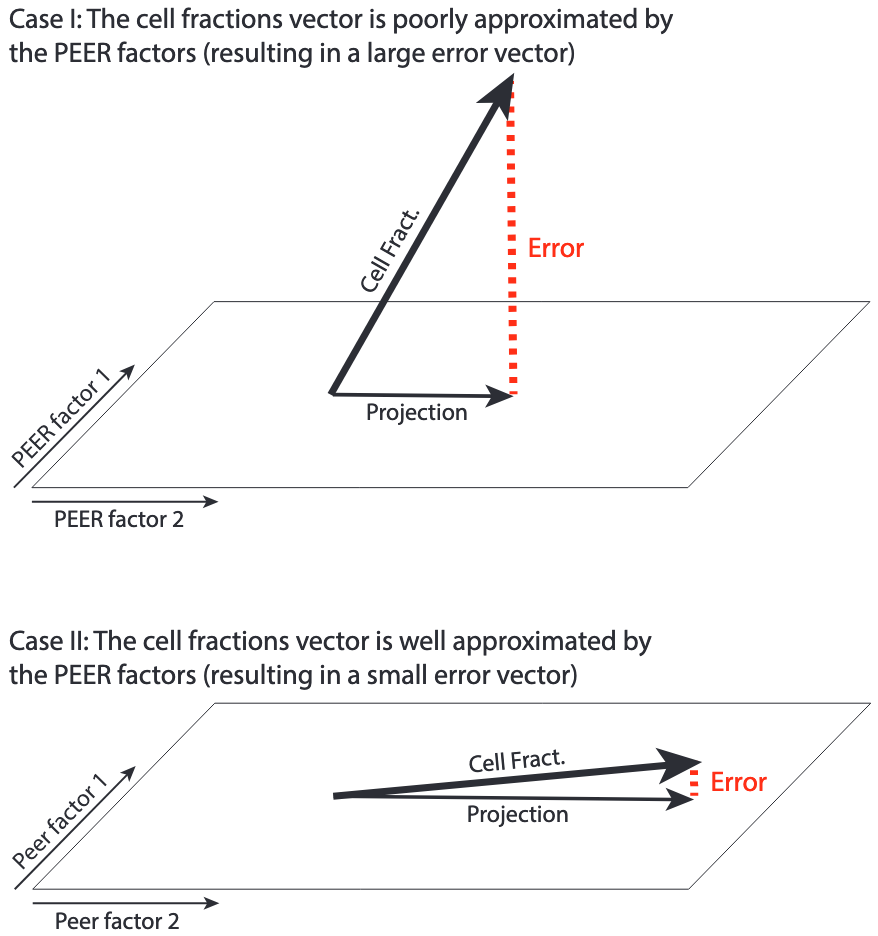


**Fig. S3.** The Cell_fract vector may lie far from the PEER space (case I) or almost entirely contained within it (case II). Errors closer to zero indicate that PEER factors more effectively incorporate information regarding cellular heterogeneity.

In addition to these geometric analyses, we also performed a series of multivariate linear regressions for each cell type. Here, we used the cell type abundance of a given cell type as the response variable, and the set of 50 PEER factors as predictive variables. The results are given in Fig. S5. Most cell types exhibit significant linear associations with the 50 PEER factors.


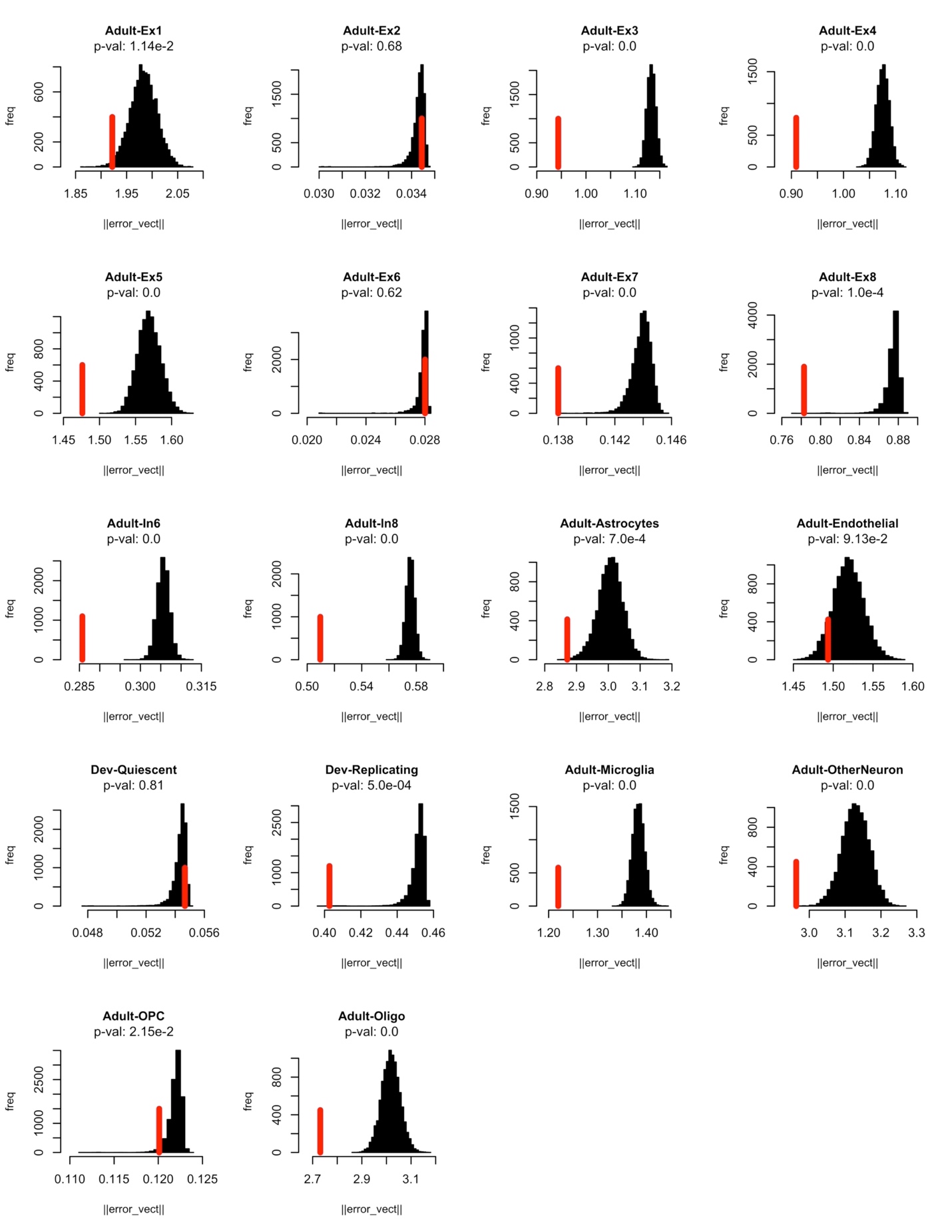


**Fig. S4.** For each cell type, we generated a null distribution using 10,000 randomly-oriented **Cell_fract** vectors, and then recorded the lengths of the associated **Error** vectors. Each p-value indicates the fraction of randomly-generated **Cell_fract** vectors that have lower associated error terms than the observed error terms (shown as vertical red lines), with this observed error term having been derived using our real (i.e., observed and non-permuted) **Cell_fract** vector.


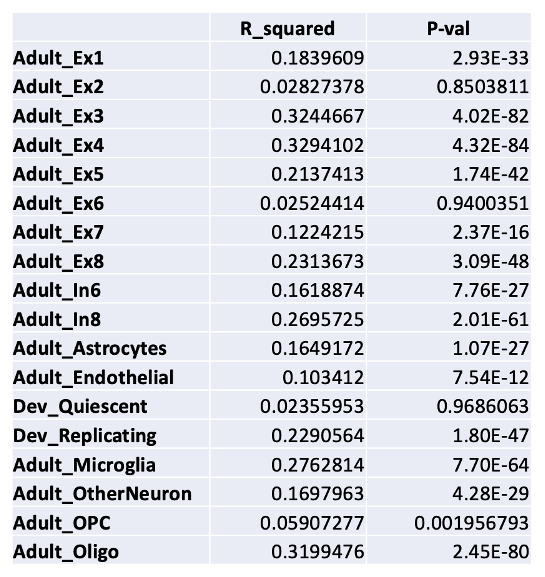


**Fig. S5.** Each cell type was used as a response variable in a linear regression on the 50 PEER factors.


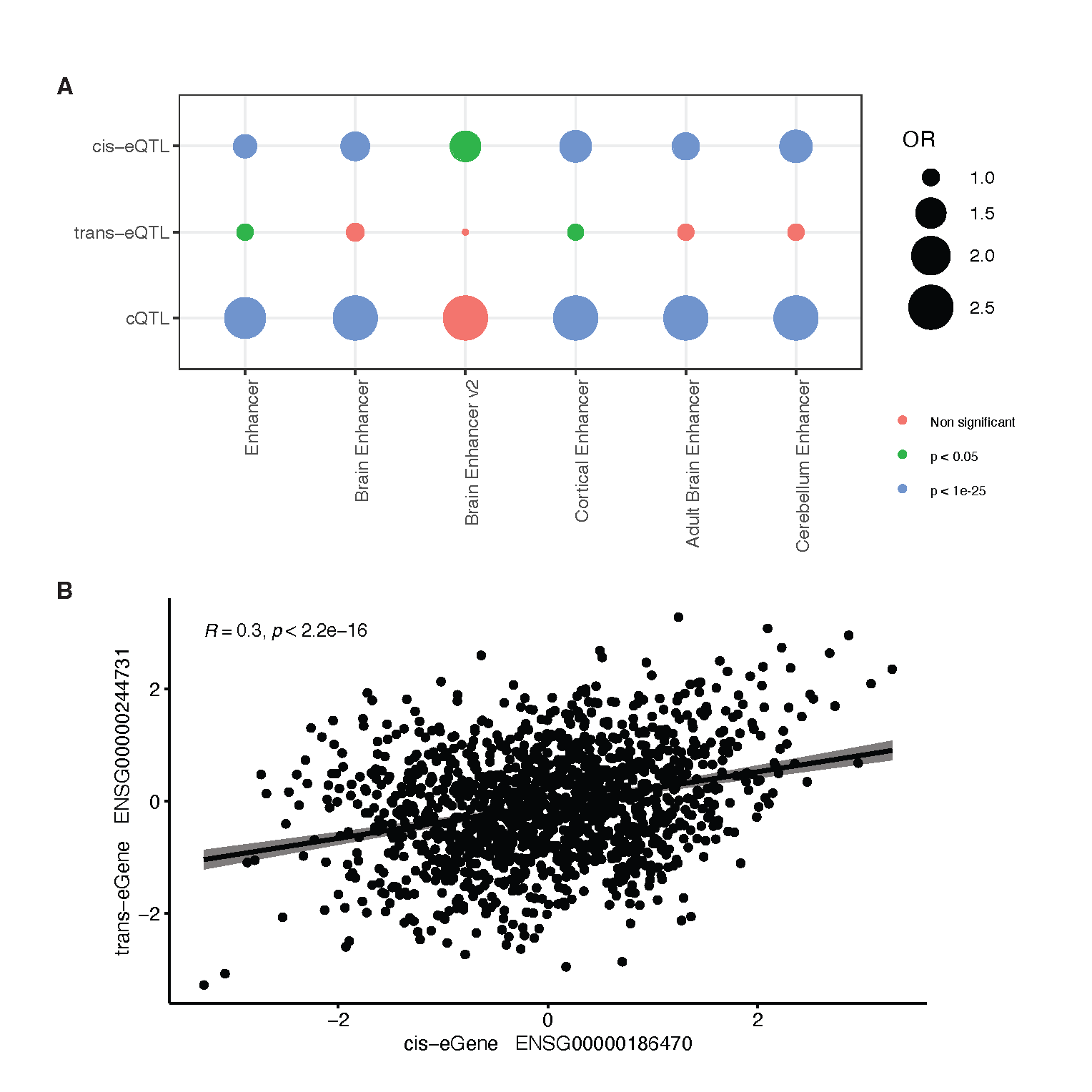


**Fig. S6. A.** Enrichment statistics of cis-eQTLs, candidate trans-eQTLs and cQTLs of different enhancer lists. The enrichment patterns are similar across different brain enhancer lists. **B.** Example for the correlation of one trans-eGene with the corresponding mediator cis-eGene sharing the same SNP in an exon.

**
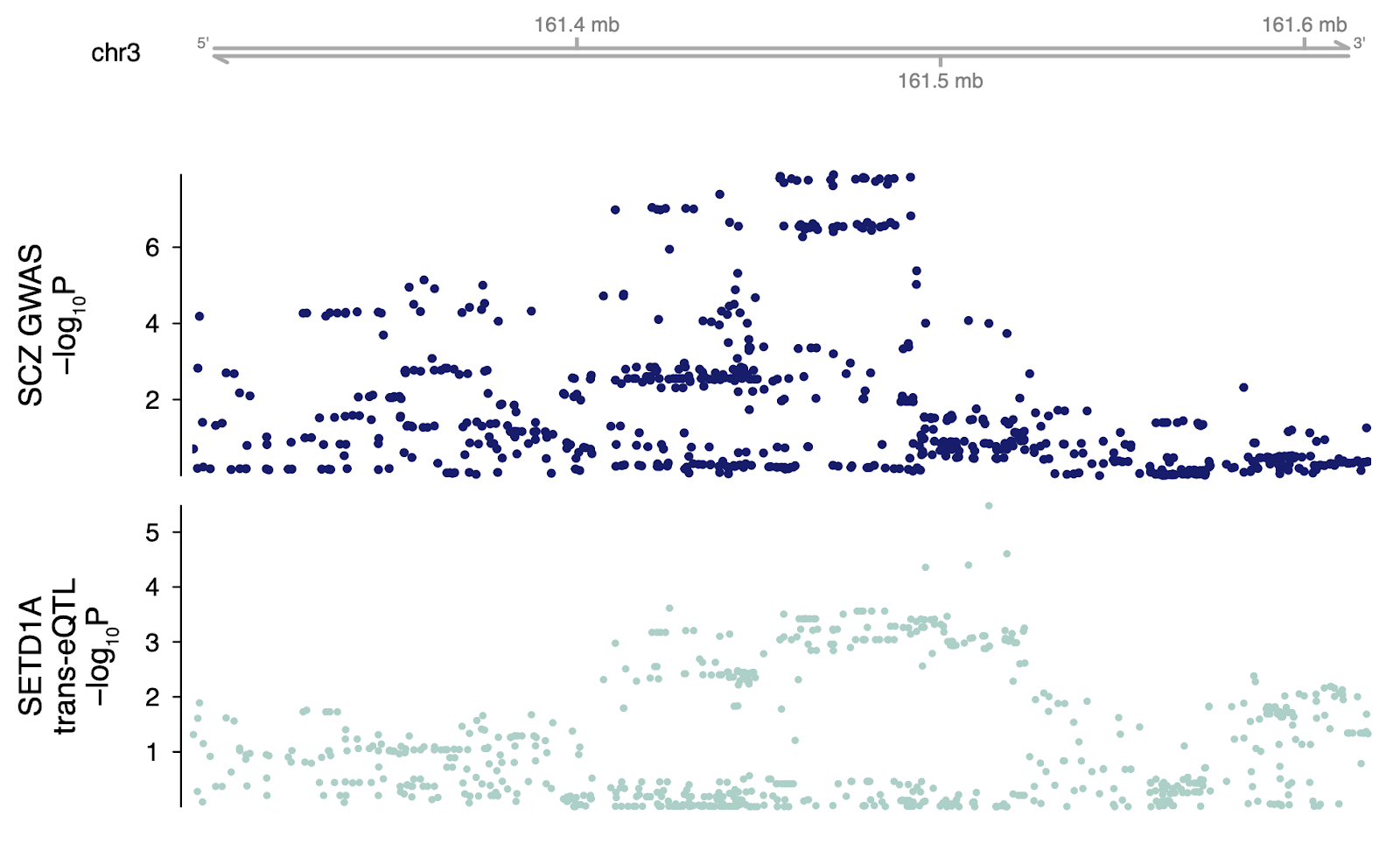
**

**Fig. S7.** A SCZ GWS locus colocalizes with candidate trans-eQTLs for *SETD1A*. The top trans-eQTL for *SETD1A* in this locus is rs17491851 (trans-eQTL P= 3.3x10^–6^, GWAS P=1x10^–4^).
